# Seed Banking as Cryopower: A Cryopolitical Account of the Work of the International Board of Plant Genetic Resources, 1973–1984

**DOI:** 10.1111/cuag.12236

**Published:** 2019-11-26

**Authors:** Sara Peres

**Keywords:** seed banks, cryopolitics, crop genetic resources, history of science, conservation

## Abstract

The implications of freezing seeds to conserve genes statically and for the long term are complex and deserve further reflection to appreciate seed banking as an attempt to detach seeds from their life cycle. Here, I use a cryopolitical framework to explore this in the context of the activities of the International Board of Plant Genetic Resources (IBPGR) between 1973 and 1984. I suggest that the emergence of seed banks is a shift toward a cryopower mode of governance, where technoscientific intervention in the biology of seeds was presented as a means to manage the survival of seeds. The project of ex situ conservation is a socio‐technical effort by international institutions such as IBPGR and a variety of institutions with seed repositories. In creating a coldscape, they sought to make genetic resources into frozen seeds that were stable and mobile, not only across space but, importantly, over time. Consequently, our interpretations of seed banks as sites of geopolitical significance in the controversies over access to seeds can be complemented by considering their biopolitical importance as interventions that extend the power of IBPGR and other institutions toward plant life, and the future.

## Introduction

Seed banks have been associated in scholarly analyses with an understanding of agrobiodiversity as useful “genetic resources” for the future. Fenzi and Bonneuil (2016) have argued that the first seed banks were organized in line with a “resourcist” international policy regime where seeds were conceptualized as global public goods, privileging the interests of plant breeders and agro‐industry. Moreover, Kloppenburg (2004) argued that seed banks were part of a process of commodification of nature and “accumulation by dispossession” with the rise of biotechnology. As an ex situ method, seed banking has been critiqued as important, yet reductionist and utilitarian, privileging the molecular scale and facilitating access for future utilization (van Dooren 2009), while cleaving plants from their biocultural environment and thus preventing the continued flourishing and evolution of plant diversity.

These analyses make a compelling case for seeds as objects of governance under the control of plant breeders and international organizations, leading to geopolitical disputes over access to and control of seed bank collections. For example, historical accounts have described how disputes between the United Nations’ Food and Agriculture Organization (FAO) and the Consultative Group on International Agricultural Research (CGIAR) shaped gene banking. For FAO, the vision for systematic genetic conservation involved the building of new seed stores as part of new Gene Exploration Centres sited in areas rich in crop genetic diversity worldwide (Busch et al. 1995; Curry 2017; Kloppenburg 2004; Pistorius 1997). Instead, CGIAR favored the conservation of priority crops in seed banks, according to the needs of breeders, and doubted FAO's expertise on technical practices of collecting and preserving seed stocks. At the 1972 Beltsville conference, CGIAR put forward the proposal that led to the establishment of the International Board on Plant Genetic Resources (IBPGR) (Frankel 1988, 22) as an “intermediary in GR [Genetic Resource] management” (Fenzi and Bonneuil 2016, 77). IBPGR was proposed as an alternative coordinator for genetic conservation[Link] and followed CGIAR's vision for seed conservation as the management of a global resource for the future. IBPGR became operational in 1973, as agro‐industry was gaining influence in genetic resources conservation (Fenzi and Bonneuil 2016). Yet, by the 1980s, the Board was criticized by diplomats from developing countries and RAFI activist Pat Mooney (Mooney 1983) for siding with corporate interests, and furthering the priorities of its developed country governmental funders over those of farmers and developing countries. Moreover, Mooney contested that “it is now clear that gene banke [sic] storage is not so secure and that the true extent of its shortcomings have been withheld from the Third World by IBPGR” (Mooney 1980, 78).

In this article, I suggest that we may deepen our understandings of the political controversies regarding the governance of seed banks by taking into account the biopolitical significance. The conceptual framework of cryopolitics is a useful complement here. Bravo and Rees (2006) originally used the term to refer to the ways in which the earth's frozen states came to be valuable in the Anthropocene (Bravo 2017, 28); it was later extended by Radin and Kowal to refer to the “tactics and practices that animate science and technology” in projects where cold temperatures are produced in order to preserve living organisms (Radin and Kowal 2017). It extends the Foucauldian concern with biopolitics of making live and letting die (Foucault 1978) to making live and *not* letting die: In other words, the power to regulate life through technoscientific means “such that death appears perpetually deferred” (Radin and Kowal 2017).

Friedreich has argued that the emergence of cryobanks in the 1970s “[gave] birth to a new mode of governance, cryopower—with refrigeration as a main *dispositif* [Link] for controlling, transforming, safeguarding, and enhancing life” (Friedrich 2017, 61). Paying attention to the strategies of seed banking as a means to control plant life into the future thus highlights the role of IBPGR and seed banking institutions as a form of cryopower.

In this article, I provide a cryopolitical account of the work of Board and, to a lesser extent, the FAO Panel of Experts on Plant Exploration and Introduction (henceforth, FAO Panel; established originally in 1967) and the CGIAR through a historical discursive analysis. I draw from policy documents and reports produced by these organizations from the period between the FAO/IBP Technical Conference in 1967 (where attendees discussed the feasibility of seed banking as a long‐term conservation tool) and 1984 (by which year renewed attention was being paid to other methods of conservation, and the FAO was calling for the formation of an alternative network of seed banks under its jurisdiction). This corpus of internal, official documents represents a particular institutional standpoint that, when read in context, provides insight into how institutions conceptualized the long‐term storage of seeds and justified strategic decisions.

IBPGR identified conservation priorities for crops and geographic regions and organized collecting missions. Most importantly for the purposes of this account, it was central to the international coordination of the infrastructure of gene banking. It established minimum standards for conservation and regeneration of seeds and other vegetative material. Additionally, it was also tasked with “arrang[ing] for replicated storage of seed and vegetative stock” (IBPGR 1975, vi). To do so, it provided grants to improve seed storage and created a World Network of Base Collections. Hence, it had significant control over long‐term storage, advising on the management of cryogenic life and coordinating the distribution of cryopower internationally.

A cryopolitical approach provides a way of thinking about the institutional and political context in which “cold storage ha[d] made possible a particular kind of insurance that turns life itself into a source of protection against death” (Radin and Kowal 2017, 9). Thus, it shows how the genebanks set up in this decade were part of a project to manage the survival of plant genetic diversity by controlling the viability of seeds through low temperature and humidity.

As the analysis of the work of IBPGR in setting standards for genebanks suggests, seed banks were imagined as spaces where the careful management of plant reproduction and seed physiology could enable a new kind of control over the temporalities of non‐human life to preserve “genetic resources” as cryogenic life. In other words, they are a “sociotechnical effort to detach organic matter from its natural life cycle” (Friedrich 2017, 61) as a form of technoscientific intervention in the future.

Consequently, seed banks can be understood as a *coldscape*: “an infrastructure, a constellation of social and technical systems that stabilize otherwise ephemeral and dynamic materials so that they can circulate, producing nutrition, comfort, health, and knowledge, albeit unevenly across the globe” (Twilley 2012 quoted in Radin and Kowal 2017, 5). In creating a World Network of Base Collections, IBPGR attempted to put the resourcist cosmovision of seed banking into practice. However, the challenges it encountered indicate that the hopes for cold storage as a means to stabilize genetic resources were only partially realized, as new questions emerged regarding the stability and vulnerability of the coldscape itself. Focusing on the governance of seed banking as a form of cryopower simultaneously focuses on the extension of control toward the future through scientific management of plant life and makes visible the contingencies and ambiguities that are implicit in these attempts to ensure their survival.

## Cryogenic Life: Defining How to Store and Replicate Seeds to Conserve Genetic Resources

Unlike other collections of plant varieties, the purpose of seed banks was to preserve the genetic constitution of samples as statically as possible, and for the long term. In this section, I describe how the FAO Panel and IBPGR envisioned seed bank infrastructure and manipulation of seed viability as a means of solving the problem of genetic erosion by creating cryogenic life.

Existing seed breeders’ working collections were considered unsuitable for systematic conservation of genetic diversity. As Otto Frankel (1900–1998), Chairman of the FAO Panel, noted, “[m]ost collections of this sort of plant material are distinctly less than effective; very few can be regarded as genebanks” (Bennett 1968; 9).^1^ Instead, he and others envisioned repositories capable of maintaining plant genetic material viable for the long term, as discussed at the 1967 FAO/IBP^2^ Technical Conference on Plant Genetic Resources.

Not all attendees agreed that seed banks were the best approach to genetic conservation (see, e.g., Bennett 1968; Frankel and Bennett 1970), debating whether the stasis of populations was desirable or even possible. One major concern was the lack of stability of samples as individually preserved seeds died, albeit at different rates. Long‐term conservation therefore required periodic “regeneration” (by re‐growing stored seeds, harvesting, and desiccating into new samples) in order to avoid death, but doing so raised the possibility of causing samples to change through genetic drift at each cycle.

For Frankel, these issues could be avoided with the appropriate storage practices, and he argued that “[a]ll of these [difficulties in maintaining collections] are mitigated by long‐term *seed storage* under optimal conditions, for it is obvious that the less a collection is exposed to the risks of life, the safer and cheaper is its maintenance” (Frankel and Bennett 1970, 482). Yet, this was, in his view, uncharted territory: There were no data on the viability of seeds under the only kind of (optimal) storage conditions that he found acceptable. Under such conditions, he thought, a large proportion of seeds could be stored with “a minimal risk of genetic damage, and regeneration should be a rare event”—even if much remained to be decided about the administration of seed banking (Frankel and Hawkes 1975, 7–8 quoted in Pistorius 1997, 51). Hence, seed banking appeared to offer the means to extend seed scientists’ control over the biology of crops into the long term through cold storage, while also enabling simultaneous study and utilization in the present (Frankel and Bennett 1970, 482).

The next step for FAO's Panel with respect to the cryopolitical strategy for seed banks was to define the organization of the coldscape. Over a series of meetings, it developed recommendations for the division of labor between collections oriented toward long‐term conservation and stability, and more immediate tasks of distribution and multiplication (Pistorius 1997, 51–52). It suggested that long‐term storage be carried out in specialist “base collections,” enabling seeds to be kept under stringent storage conditions and regenerated at the most appropriate time. In turn, they would only be accessed when required to replenish the “stock” of samples in “active collections” that carried out medium‐term storage, regeneration, multiplication, and distribution, evaluation, and documentation of genetic resources (FAO 1975, 31). The coldscape was envisioned as collaborative and international, in line with the resourcist idea of seeds as global public goods.

IBPGR's activities were important in defining the apparatus of cryopower by providing guidance and setting standards for genebanks. It established a Working Group on Seed Storage, headed by plant scientist and panel member, E.H. Roberts, to develop guidance about infrastructure and practice standards for base collections (e.g., IBPGR 1975, b). With respect to ensuring long‐term maintenance, it adopted the panel's recommendations for preferred and acceptable conditions at base collections (IBPGR 1977b). To achieve “preferred conditions,” the panel recommended drying seeds to circa 5% moisture and storing in airtight containers at −18°C minimum. Less stringent conditions of 5°C or less in situations where humidity was controlled (either with or without sealed containers) were considered “acceptable” (Crop Ecology and Genetic Resources Unit 1975).

The Working Group also prescribed that seed viability be monitored, and samples replicated as soon as viability decreased five to ten percent (IBPGR 1977a,b), emphasizing that regeneration itself was costly and incurred “difficulties and dangers,” not least of which were “loss of purity… through mechanical mixing and cross‐pollination, and in generically heterogeneous samples, selection [which would change the genetic composition of the accession]” (IBPGR 1977b).

Finally, it commissioned research into seed physiology and the relationship between storage conditions and seed viability. Roberts's Seed Science Laboratory at the University of Reading (UK) received grants totaling 289,310 USD between 1977 and 1981 (see Table 1 for yearly amounts) to produce plots of survival curves on which IBPGR could recommend the frequency of monitoring tests (IBPGR 1978, 39; published as Ng and Williams 1979). The rationale for this work was explicitly related to the need to address ambiguity about seed viability. As IBPGR put it, “such [regeneration] intervals must not be too frequent, or else the stocks will become depleted, but must be frequent enough to assure the early detection of loss of viability” (IBPGR 1980, 60).

**Table 1:** Grants (in USD) Dispensed Annually by IBPGR Between 1976 and 1985, in Relation to its Total Expenditure

	Grants under “conservation” (where this included research grants, they were subtracted and noted in the next column) (USD)	Grants for research (USD) (* = tallied under “conservation”). [Amount granted to the University of Reading]	Total expenditure (USD, to nearest dollar)	Reference
1976	N/A (but projected budget of 468,000 to all crop exploration activities in 1975 Annual Report, pp. 20 and 21)	N/A	914,833	IBPGR/76/27, p. 31
1977	144,207	[22,581]*	1,285,005	IBPGR/78/8, pp. 63, 66
1978	180,000	[49,151]*	1,715,401	IBPGR/79/8, pp. 85, 89
1979	110,000	125,744* [53,976]	2,369,216	IBPGR/80/5, pp. 89, 93–94
1980	180,956	74,063* [20,000]	3,554,645	IBPGR/81/24, pp. 93, 99–1000
1981	308,100	165,194* [143,602]	4,451,809	IBPGR/82/19, pp. 101,105–107
1982	N/A	N/A	4,720,301	IBPGR/83/40, p. 102
1983	N/A	N/A	4,510,152	IBPGR/84/61, p. 111
1984	527,781 (“Conservation”)	215,562 (“Strategic Research”)	4,338,825	IBPGR/85/71, p. 105
1985	805,244 (“Global Genetic Resources Network—includes regional coordination”)	255,333 (“Seed Conservation Research”)	5,562,479	IBPGR. 1986. Annual Report, 1985. p. 82

As this account illustrates, the idea of preserving seeds through freezing required the production of new scientific knowledge and the devising of an infrastructure of seed banking able to provide “an oscillation between various states” (van Dooren 2017, 264) in order to stabilize seeds—thus, creating cryogenic life. Through its standard‐setting work, IBPGR became a focal point for the cryopolitics of seed banking. In addition to defining what appropriate seed management was, it was also engaged in coordinating and structuring the coldscape as it determined which institutions could be trusted to properly care for these base collections and would therefore receive resources for this purpose.

## Cryopower: IBPGR's Role in “Catalyzing” the Coldscape of Genetic Conservation

Another priority aim of the IBPGR during this decade was to encourage and support the development of long‐term seed stores worldwide. To do so, it provided funding for equipment and seed store building and organized the World Network of Base Collections, a group of international, regional, and national institutions invited to “accept responsibility for holding ‘world’ or ‘regional’ base collections of some important crops” (IBPGR 1978, 38). I describe its work up to 1984 and argue that the production of long‐term seed stores reproduced and enacted the resourcist framing of seeds as organisms that could be managed, stabilized, and shared. However, the technical and political developments of the 1980s challenged the assumptions built into the coldscape.

The construction of the coldscape reflected largely CGIAR's strategic focus on “useful” germplasm for the Green Revolution, rather than FAO's vision of systematic conservation (Pistorius 1997: 60). Although both called for the concentration of collections in a few repositories, FAO proposed to set up new genetic resources centers in areas of high diversity. In contrast, CGIAR insisted on inviting existing institutions (including its own International Agricultural Research Centres [IARCs]) to become “base collections” if they were compatible with the newly defined standards, and provide seed to various active collections associated with it (IBPGR 1978, 66; see also Busch et al. 1995, 50). The cryopolitical logic of conservation meant that securing crops was a matter of developing a seed banking infrastructure that met IBPGR's requirements, regardless of location.

Pistorius suggests that CGIAR's “strong financial backing (…) contributed to a pragmatic approach” because it was the only organization providing international financial support for seed banks (Pistorius 1997, 62). The funding available to IBPGR was not, however, sufficient to create a whole new network. Indeed, as Table 1 (below) demonstrates, the funds disbursed were relatively modest. The board repeatedly noted that it was not a long‐term funder. It could “promote, encourage, initiate and help but could not (with any conceivable funding) itself build and sustain the genetic resources conservation network” (IBPGR 1981, 76). Instead, it characterized itself as a “catalyst,” and its financing limited to “capital projects” and “urgent collections” (IBPGR 1977a, 1), “intended to support the work of institutions and individuals everywhere on plant genetic resources.”

IBPGR's approach to funding thus resembled prime‐pumping investments: Time‐limited injections of capital intended to fund future value production. It allocated funds to “priority” regions or crops where it might best benefit users (plant breeders) and only distributed funds as far as required to bridge the gap between the public interest and that of potential users. This suggests an underlying belief that other actors would step in to support the coldscape.

In practice, IBPGR focused on funding the building or upgrading of seed stores and the provision of equipment, thus seeking long‐term impact without a long‐term financial commitment. It allocated grants to existing genebanks to ensure enough suitable preservation capacity in centers of the network (IBPGR 1976, 2–3) by paying for upgrades or the construction of new facilities (IBPGR 1977a, 2) to increase the number of seed stores that could reach the new standards. Paradoxically, then, cryopower was distributed globally to institutions willing and able to operate in accordance with IBPGR standards for the long term, without the option of long‐term financial support from the organization.

Nonetheless, IBGPR presided over a large increase in storage capacity by engaging with existing initiatives. In 1976, it began issuing invitations to collections to join its network of base collections. Table 2 (below) provides an overview of the base collections that were added to the network between 1977 and 1985. Some were International Agricultural Research Centres (storing rice, beans, and millets), and others were national collections (e.g., the National Seed Storage Laboratory in the United States, the Vavilov Institute of Plant Industry in Leningrad, or the Plant Germplasm Institute in Kyoto, Japan). By 1977, the network had “started to take shape.” New base collections included, among others, African rice at the International Institute of Tropical Agriculture (IITA, Nigeria), cereal wild species *Triticum* and *Aegilops* at the Plant Germplasm Institute (Kyoto), oats at the Canadian Genebank and pulses at the International Crops Research Institute for the Semi‐Arid Tropics (ICRISAT) in Hyderabad, India. The network of base collections continued to grow, albeit slowly, in 1978 and 1979. IBGPR contributed to the International Maize and Wheat Improvement Center's (CIMMYT) new genebank, and it paid $67,830 “for regional storage facilities for the SE Asian Regional Programme” to a vegetable base collection at the Institute of Plant Breeding (Los Baños, Philippines) and $25,000 to IITA in Ibadan, Nigeria (IBPGR 1978, 66). As the list indicates, IARCs were often beneficiaries from this program. Other base collections were, more often than not, located in developed countries. When located in developing countries, they were bankrolled by contributions from richer nations, as was the case with two new Genetic Resources Centres in Costa Rica and Ethiopia, funded by West Germany. Thus, the network was aligned with centers of agricultural research and development. It is, however, worth emphasizing the role of expertise, availability of funding for the future, and willingness to subscribe to the resourcist paradigm of agrobiodiversity.

**Table 2:** Base Collections in the IBPGR World Network, Including Year First Established for that Crop and Allocated Base Collections in the Network

Crop	Base collections
Barley	1980: Canadian Genebank (global collection), NGB, Sweden (European collection), PGRC/E, Addis Ababa, Ethiopia (African collection)
Maize	1977: NSSL, USA (“new world”), NIAS, Japan (“Asian”); VIR, USSR (European); 1980: Portuguese Genebank, Portugal (Southern European)
Millets	1977: PGR, Canada; ICRISAT, India; NSSL, USA; PGRC/E, Ethiopia; NBPGR, India
Oats	1977: PGR, Canada; 1981: NGB, Sweden
Rice	1977: IRRI, Philippines; NIAS, Japan; IITA, Nigeria; NSSL, USA
Rye	1981: Polish Genebank; NGB, Sweden (global)
Sorghum	1981: NSSL, USA; ICRISAT, India
Wheat	1977: VIR, USSR; CNR, Italy; NSSL, USA; PGI, Japan
Chickpea	1977: ICRISAT, India (global)
Faba bean	1984: CNR, Italy (global)
Groundnut	1977: ICRISAT, India (global); 1980: INTA, Argentina (South American); CENARGEN/EMBRAPA (wild perennial species)
Lupin	1982: ZIGuK, GDR; INIA, Spain (European)
Pea	1979: NGB, Sweden (global); CNR, Italy (Mediterranean); Polish Genebank (Central and East European)
*Phaseolus*	1977: CIAT, Colombia (duplicated to NSSL, USA in 1978); 1979: Faculté des Sciences Agronomiques de l’État, Gembloux, Belgium; FAL, Germany, FR (European)
Pigeon pea	1977: ICRISAT, India
Soybean	1983: NSSL, USA; CSIRO, Australia (wild perennial)
*Vigna* spp	1983: Faculté des Sciences Agronomiques de l’État, Gembloux, Belgium; IBP, Philippines; AVRDC, China; IITA, Nigeria; NSSL, USA
Winged bean	1981: IBP, Philippines; TISTR, Thailand
Cassava	1983: CIAT, Colombia
Potato	1980: CIP, Peru
Sweet potato	1983: NSSL, USA (global); AVDRC, China (Asian); NIAS, Japan
*Allium*	1981: NVRS, UK; NSSL, USA; RCA, Hungary (South and East European); NIAS, Japan (Asian)
*Amaranthus*	1981: NSSL, USA (global); NBPGR, India (Asian)
Capsicum	1981: CATIE, Costa Rica; IVT, the Netherlands
Crucifers	1981: FAL, Germany, F. R.; Canadian Genebank; PGRC/E, Ethiopia; NVRS, UK; Universidad Politécnica, Madrid, Spain; Tohoku University, Sendai, Japan; PGR; NIAS, Japan
Cucurbits	1983: IPB, Philippines; NSSL, USA; INIA, Spain
Eggplant	1981: IVT, the Netherlands; NSSL, USA
Okra	1983: NSSL, USA
Tomato	1981: CATIE, Costa Rica; ZIGuK, GDR; NSSL, USA (global); IBP, Philippines (Asian)
Southeast Asian vegetables	1977: IPB, Philippines
Sugar beet	1977: FAL, Germany, Federal Republic; 1982: NGB, Sweden
Cotton	1985: Greek Genebank
Sugarcane	1984: NSSL, USA
Tobacco	1985: Greek Genebank
Tree species	1984: RBG, UK

Please see legend for acronyms below. When these are an International Agricultural Research Centre of the CGIAR, this is indicated by IARC. All others are national collections taking on international responsibilities. I have noted where collections are global versus regional. However, for clarity, I am only reporting the crops held even where there are two more different types at one institution (e.g., different types of Brassica at NVRS, UK).

This table is based on data collected from the following documents: IBPGR/1978/8, p. 38; IBPGR/79/8, p. 55–56; IBPGR/80/5 p. 58; IBPGR/81/24 pp. 58–59; IBPGR/82/19, pp. 66–67, IBPGR Annual Report 1985, pp. 90–91.

AVRDC, Asian Vegetable Research and Development Center, China; CATIE, Centro Agronómico Tropical de Investigación y Enseñanza, Turrialba, Costa Rica; CIAT, Centro Internacional de Agricultura Tropical, Colombia (IARC); CIP, Centro Internacional de la Papa, Peru (IARC); CNR, Consiglio Nazionale delle Ricerche, Bari (Italy); CSIRO, Commonwealth Scientific and Industrial Research Organization, Australia; EMBRAPA/CENARGEN, Empresa Brasileira de Pesquisa Agropecuária/Centro Nacional de Recursos Genéticos, Brazil; FAL, lnstitut für Pflanzenbau und Pflanzenzuchtung der Bundesforschungsanstalt für Landwirtschaft, Branschweig, Germany, FR; ICRISAT, International Crops Research Institute for the Semi‐Arid Tropics, India (IARC); IITA, International Institute of Tropical Agriculture, Nigeria (IARC); INIA, Institute Nacional de Investigaciones Agrarias Spain; INTA, Instituto Nacional de Tecnologia Agropecuaria, Argentina; IPB, Institute of Plant Breeding, (Philippines); IRRI, International Rice Research Institute, (Philippines, IARC); IVT, Institute for Horticultural Plant Breeding Wageningen, the Netherlands; NBPGR, National Bureau of Plant Genetic Resources, India; NGB, Nordic Genebank, Sweden; NIAS, National Institute of Agricultural Sciences, Japan; NSSL, National Seed Storage Collection, USA; NVRS, National Vegetable Research Station, Wellesbourne, UK; PGI, Plant Germplasm Institute, Kyoto, Japan; PGRC/E, Plant Genetic Resources Center, Ethiopia; RBG, Royal Botanic Gardens, Kew (UK); RCA, Institute for Plant Production and Qualification, Hungary; TISTR, Thailand Institute of Scientific and Technical Research, Thailand; VIR, N.I. Vavilov Institute of Plant Industry, USSR; ZIGuK, Zentralinstitut für Genetik und Kulturpflanzenforschung, German Dem. Rep.

The board expected institutions taking part in the network (and/or receiving funding from IBPGR) to uphold “principles” of collaboration and exchange across borders. IBPGR favored the creation of a network of co‐operating institutions that shared responsibilities for conservation across national borders. It was particularly interested in funding collections willing to take up regional responsibilities, allocating $65,000 to the INIA in Madrid in 1978 “with an understanding that it will store grain legumes and possibly other crops on a regional basis as well as acting as a national repository for all crops” (IBPGR 1980, 56). Repositories in the network were expected to “freely exchange both genetic materials and information related to them” (IBPGR 1976). They were intended as regional nodes for preservation, engaging in the collecting of germplasm across the area and supplying multiple countries, therefore ensuring cost efficiency and a “rational” system. Each base collection should also be replicated in at least two different institutions for safety, and IBPGR mandated that collecting expeditions left duplicate samples in their countries of origin.

However, by the end of the decade there was increasing recognition that seed banks were encountering technical challenges, or simply had no resources to comply with IBPGR's expectations. First, collections were not safety duplicated elsewhere due to insufficient storage capacity (IBPGR 1981, 51). Neither were all seed banks multiplying seeds adequately or frequently enough, in turn limiting both regeneration and the availability of seeds to users. Here, funding and small sample size were “frequently a constraint in making the duplication and exchange effective” (IBPGR 1982a, 67). In response, IBPGR commissioned certain seed banks to multiply collections on behalf of others. In 1981, it allocated funds to banks in France ($8000) and Ottawa ($5000) to multiply pearl millet samples from Africa, and to the Netherlands ($10,000) for material from Ethiopia and Pakistan, among others (IBPGR 1982a, 67–68).

Moreover, the board convened an ad hoc International Advisory Committee on Seed Storage in September 1981 (again chaired by E. H. Roberts), partially to address practical issues found by curators (IBPGR 1982b, 1984b). It reported that “in many cases seeds were not being stored under the most appropriate conditions to ensure optimum longevity” (IBPGR 1984b, 1). Other “constraints” to viability were found—from transport issues to lack of information about samples and problems during the original storage process. Some collections did not carry out regular seed testing and viability monitoring regimes, while in others there were delays to the conservation or distribution of samples because of sample backlogs and quarantines.

The original guidance for the management of cryogenic life over time in well‐coordinated, supranational collections was therefore proving to be difficult. At this time, IBPGR seemed to re‐scale its focus toward repositories that were smaller, more affordable, and often national in scope. In 1982, the Advisory Committee on Seed Storage published a revision of its original 1976 report in light of “a number of scientific and technical developments in seed storage” and changes in “economic circumstances (…) since 1976.” For instance, it updated its recommendation for preferred temperature storage of −18°C in 1976 (Cromarty, Ellis, and Roberts 1982, 2) to facilitate the use of commercially available freezers for storing small seed collections, rather than expensive, large specialist units (Ellis and Roberts 1982).

Much was undoubtedly achieved in a relatively short time: By 1983, 30 designated base collections existed in 24 countries, of which, the Annual Report noted, half were in developing countries—perhaps in response to the criticism of IBPGR for the placing of seed banks in the Global North. Indeed, the board expected to “form a reasonably complete network” by 1986, with 50 base collections for 40 crops (IBPGR 1984a, 77). However, not all collections managed to reach IBPGR standards; one 1984 report put this number at 55 out of approximately 100 (IBPGR 1984b, 3). Indeed, by 1984 IBPGR announced its policy to “discontinue funding for the construction of new long‐term seed stores (except in exceptional cases)” altogether, although it would continue to upgrade already existing ones (IBPGR, 1985, 64). It also stepped back from the “widespread collecting of cultivars” with collections only in case of documented emergency situations—focusing instead on “characterization, documentation and generally bringing order to existing collections” (IBPGR 1984a, vii).

IBPGR's organization of cryopower, then, was intended to produce a coldscape that, like the resources it sought to maintain, was global, efficient, stable, and uniform. However, while the network was intended as a means to stabilize and protect genetic resources, the large, international base collections that were central to IBPGR's vision were themselves vulnerable to difficulties over the long term. An increasing recognition of the technical and political challenges posed by base collections in the 1980s contrasts IBPGR's vision for cryopower in a resourcist context, and the more partial, contingent, and spatialized network of seed banks that resulted by the end of the decade.

## Conclusions

My account of IBPGR's activities between 1973 and 1984 suggests that the emergence of seed banks in the 1970s represents a shift toward a particular mode of governance of seeds that Friedrich (2017) has termed “cryopower,” where freezing was deployed to manage the survival of seeds. From this angle, the project of ex situ conservation in seed banks was a socio‐technical effort by institutions including IBPGR, FAO, CGIAR, and variety of institutions with seed repositories. In creating a coldscape, they sought to make genetic resources into frozen seeds that were stable and mobile, not only across space but, importantly, over time. Consequently, our interpretations of seed banks as sites of geopolitical significance in the controversies over access to seeds can be complemented by considering them as biopolitical interventions that extend the power of the IBPGR, FAO, and CGIAR into non‐human life, and toward the future. Understanding the planning and functioning of the apparatus of cryopower enables us to explore seed banks as sites with a very specific function—separating the seed from its natural life cycle and temporal orientation by saving seeds (which have been endangered by agricultural modernization) so that they may serve as future gene donors.

A cryopolitical framework examines how technoscientific practices and tactics were deployed to avoid genetic erosion by creating seeds as cryogenic life, but also questions the “cold optimism” that it is possible to postpone death indefinitely through cold storage (Radin and Kowal 2017, 9). As the IBPGR standards demonstrate, conserving genes required careful reproduction and management of seed samples in order to detach them from their natural life cycle to minimize genetic changes over time. Indeed, IBPGR's guidance for viability maintenance, and the need to revise it in the 1980s, further underlines the difficulties involved in creating and maintaining cryogenic life over time. It suggests that the hopeful horizon of technoscientific salvation was in tension with the capacity of some collections to carry out the work required of IBPGR in the present.

This account simultaneously draws our attention to seed banking as a move to control plant life and invites us to probe its limitations. It suggests that seed banking is a contingent process where curators preserve and manipulate biological materials through freezing, monitoring, and regeneration. Therefore, the continued maintenance of seed bank collections deserves scholarly attention, in parallel with the histories of plant exploration. Further work is required to describe the myriad institutional, economic, and biological factors that influence the relative security or vulnerability of germplasm, from monitoring seeds and freezers to the stability of storage conditions and funding policies.

The establishment of the World Network of Base Collections is an example of IBPGR's work toward enacting the resourcist framing of seeds (Fenzi and Bonneuil 2016) as resources through the coldscape. Building on the CGIAR's vision for co‐situating seed banking within existing agricultural research institutions, it coordinated base collections at a global or regional scale. Its network privileged collections in specialized, supranational spaces where they could be sheltered from the dangers of genetic erosion and, in turn, remain available as potential sources of adaptation for future agricultural crops. It also allocated responsibility for base collections to institutions that were able to reach its standards and willing to manage and distribute seeds as “public goods,” in accordance with its principles. In this way, the distribution of cryopower tracked fairly closely and reinforced, the existing framing of the Global South as providers of genetic diversity, and of technoscientific intervention as the solution to the problem of genetic erosion.

Indeed, this finding suggests another political implication of seed banking, beyond their role in agroindustrial exploration of genetic material from “gene‐rich” countries. Given that the kinds of futures envisioned and facilitated by the coldscape were, by dint of the very apparatus of cryopower, geared toward the interests of plant breeders over those of farmers, those unable to access or engage with the coldscape were therefore doubly excluded: not only from their rights to be acknowledged or recompensed as owners of the seed, but also from the ability to influence decisions related to the future constitution of crop genetic diversity. From this perspective, then, the Seed Wars (e.g., Aoki 2008) are also, fundamentally, cryopolitical in nature.

Yet, this account also points to optimism regarding the recognition of the benefits provided by seed banks and the subsequent enrollment of other actors in the project of maintaining genetic resources. The IBPGR's own “catalyst” approach to funding meant that the long‐term responsibility for the survival of individual base collections was devolved to the host repositories. Here, too, there is a tension between the hope of making seeds not die in seed banks and the suggestions that the future of seed banks themselves is not necessarily assured. It reverberates with more recent developments with the creation of the Svalbard Global Seed Vault as the most recent attempt to simultaneously ensure the future of the coldscape and of seeds.

Altogether, contemplating the cryopolitical nature of seed banks provides a particular sensitivity to questions of survival, inviting us to contemplate the threads connecting the future of crops with broader framings of environmental threat, development, and ultimately the sorts of futures that are considered possible with technoscientific interventions like freezing seeds.

## Notes

1 IBPGR was an autonomous organization “under the aegis of the CGIAR” but hosted at, and with secretariat assistance from, FAO's Crop Ecology and Genetic Resources Unit. After 1991, it officially became part of the CGIAR and the name was changed to the International Plant Genetic Resources Institute.

2 This Foucauldian term refers to an apparatus or “system of relations” composed of a heterogeneous constellation of “discourses, institutions, architectural forms, regulatory decisions,” and many other elements. It can be thought of as a program of action or an application of a specific practice to a new field of rationality in order to address a specific, urgent need (Foucault and Gordon 1980).
